# Risk factors of hypertension in South Africa: A systematic review and meta-analysis to inform the development of a clinical prediction model

**DOI:** 10.4102/hsag.v31i0.3385

**Published:** 2026-09-25

**Authors:** Martins Nweke, Julian Pillay

**Affiliations:** 1 Department of Basic Medical Sciences, Faculty of Health Sciences, Durban University of Technology, Durban, South Africa

**Keywords:** hypertension, risk factors, South Africa, systematic review, meta-analysis, prediction model, clinical prediction

## Abstract

**Background:**

Hypertension is a major cause of preventable cardiovascular morbidity and mortality and remains a significant public health challenge in South Africa. Most existing risk prediction models were derived from high-income populations and may not perform well in low- and middle-income settings.

**Aim:**

To synthesise pooled evidence on risk factors of hypertension in South Africa and use the findings to inform the development of a clinical prediction model.

**Setting:**

South Africa.

**Methods:**

A systematic review and meta-analysis were conducted using studies retrieved from PubMed, Scopus, Academic Search Complete, MEDLINE, Web of Science and African Journals (SABINET) from 1990 to March 2025. Titles, abstracts and full texts were screened independently by two reviewers. Random-effects meta-analysis was used to pool odds ratios for eligible risk factors. Certainty of evidence was assessed using Grading of Recommendations Assessment, Development and Evaluation (GRADE), while predictor selection for model development was guided by risk weight and geotemporal trend.

**Results:**

Fifty-four studies, including cross-sectional, case–control and cohort designs, were included. The studies covered multiple South African provinces, with 15 national studies spanning all nine provinces. Strong pooled associations were observed for dyslipidaemia (odds ratio [OR] 3.79, 95% confidence interval [CI] 1.84–7.80), age > 50 years (OR 3.49, 95% CI 1.82–6.70), diabetes (OR 2.57, 95% CI 1.86–3.53), obesity (OR 2.35, 95% CI 1.89–2.91) and gold mine dust exposure (OR 2.35, 95% CI 1.70–3.25). These findings informed the final prediction model.

**Conclusion:**

Age, metabolic factors, environmental exposures and selected social determinants were important predictors of hypertension. The resulting model may support early screening in primary care, although external validation is required.

**Contribution:**

This study synthesises South African evidence on hypertension risk factors and translates the findings into a context-specific clinical prediction model.

## Introduction

Hypertension remains one of the most important modifiable risk factors for cardiovascular disease and continues to contribute substantially to global morbidity and mortality (Forouzanfar et al. 2017:165–174; Global Burden of Disease [GBD] 2019 risk Factors Collaborators 2020:1223–1249; Roth et al. 2020:2982–3021). Elevated blood pressure is strongly associated with adverse outcomes such as stroke, coronary artery disease, heart failure and chronic kidney disease, making it a major target for prevention strategies worldwide (Forouzanfar et al. 2017:165–174; Roth et al. 2020:2982–3021). Global analyses from the Global Burden of Disease study consistently identify high systolic blood pressure among the leading contributors to disability-adjusted life years and premature mortality across both developed and developing regions (GBD 2019 Risk Factors Collaborators 2020:1223–1249). Despite improvements in detection and treatment in high-income countries, the global burden of hypertension remains high and continues to increase in many parts of the world (Mills, Stefanescu & He 2020:223–237; NCD Risk Factor Collaboration 2021:957–980).

The rise in hypertension prevalence has been particularly pronounced in low- and middle-income countries, including those in sub-Saharan Africa (Ataklte et al. 2015:291–298; Ibrahim & Damasceno 2012:611–619; NCD Risk Factor Collaboration 2021:957–980). Large pooled analyses and epidemiological investigations suggest that many countries in this region are undergoing rapid demographic and epidemiological transitions characterised by urbanisation, ageing populations and changes in lifestyle behaviours (Ibrahim & Damasceno 2012:611–619; Mills et al. 2020:223–237). As a result, hypertension prevalence has increased substantially while awareness, treatment and control rates remain comparatively low (Ataklte et al. 2015:291–298; Kayima et al. 2013:54–64). This disparity may be attributed to inadequate funding and structuring of healthcare systems in many low- and middle-income countries, particularly the limited preventive healthcare approaches that rely predominantly on biological or treatment-focused models rather than comprehensive population-level prevention strategies (Geldsetzer et al. 2019:652–662; Marmot et al. 2008:1661–1669).

South Africa reflects many of these regional patterns and faces a considerable burden of hypertension and cardiovascular disease (Addo, Smeeth & Leon 2007:1012–1018; Seedat, Rayner & Veriava 2014:288–294).

Hypertension is widely recognised as a major contributor to stroke and other cardiovascular conditions in the country, with prevalence estimates varying across provinces and population groups (Seedat et al. 2014:288–294). Several epidemiological studies conducted in African populations have identified multiple determinants associated with hypertension, including increasing age, obesity, diabetes, alcohol consumption, smoking, dietary patterns and socio-economic factors (Adeloye et al. 2015:230–242; Mills et al. 2020:223–237). Socio-economic determinants such as education, employment and income also influence cardiovascular risk through behavioural pathways and access to healthcare resources (Kaplan & Keil 1993:1973–1998; Stringhini et al. 2017:1229–1237).

A growing number of primary studies have examined hypertension risk factors using cross-sectional, case–control and cohort designs (Euser et al. 2009:214–217; Schlesselman 1982:1–354). However, these studies vary considerably in study design, sample size, measurement methods and statistical adjustments. Such heterogeneity makes it difficult to determine the relative importance of individual predictors when studies are interpreted independently. Systematic reviews and meta-analyses provide an important methodological approach for synthesising epidemiological evidence and generating more reliable pooled estimates of associations across heterogeneous studies (Munn et al. 2018:5; Page et al. 2021:71).

Globally, several systematic reviews and pooled analyses have examined hypertension prevalence and associated determinants across populations (GBD 2019 Risk Factors Collaborators 2020:1223–1249; Kayima et al. 2013:54–64; NCD Risk Factor Collaboration 2021:957–980). For example, the NCD Risk Factor Collaboration analysed global trends in hypertension prevalence using more than one thousand population-based studies, demonstrating major regional disparities in hypertension burden (NCD Risk Factor Collaboration 2021:957–980). Similarly, systematic reviews conducted in African populations have highlighted persistent gaps in hypertension awareness, treatment and control across the continent (Kayima et al. 2013:54–64). Although these investigations provide important insights into the global epidemiology of hypertension, relatively few systematic reviews have synthesised evidence specifically on hypertension risk factors within the South African context.

Beyond identifying individual risk factors, there is increasing interest in integrating epidemiological evidence into predictive frameworks that can guide prevention strategies and clinical decision-making. Clinical prediction models typically combine multiple demographic, behavioural and metabolic predictors to estimate an individual’s probability of developing disease. However, many widely used cardiovascular risk prediction models were developed using data from high-income populations and may not perform optimally in settings with different socio-economic, environmental and health system contexts.

Epidemiological theory suggests that chronic diseases such as hypertension arise from combinations of interacting component causes rather than single isolated exposures (Rothman 1976:587–592). Rothman’s causal pie model provides a useful framework for understanding how multiple risk factors combine to produce disease outcomes (Rothman 1976:587–592). Similarly, Hill’s principles of causation emphasise the importance of strength, consistency, biological plausibility and temporality when interpreting associations between exposures and disease outcomes (Hill 1965:295–300). Integrating these conceptual frameworks into evidence synthesis may therefore improve the identification and prioritisation of clinically meaningful predictors.

Systematic synthesis of hypertension risk factors in South Africa therefore provides an opportunity to strengthen the evidence base for risk prediction and prevention strategies within the country. Meta-analysis enables the combination of effect estimates from multiple studies, thereby improving statistical precision and allowing exploration of heterogeneity across populations and study designs (Page et al. 2021:71). When combined with structured evaluation of evidentiary certainty, such synthesis can help identify predictors that are both epidemiologically robust and clinically relevant. Therefore, this study aimed to synthesise pooled evidence on risk factors associated with hypertension in South Africa through a systematic review and meta-analysis and to use these findings to inform the development of a clinical prediction model. By integrating pooled epidemiological evidence with conceptual frameworks of causation and evidentiary certainty, the study seeks to contribute to improved identification of high-risk populations and support more effective hypertension prevention strategies in South Africa.

## Research methods and design

This study was a systematic review and meta-analysis conducted to synthesise evidence on risk factors of hypertension in South Africa and to inform the development of a clinical prediction model. The review followed the Preferred Reporting Items for Systematic Reviews and Meta-Analyses (PRISMA 2020) guidelines and was also informed by the Transparent Reporting of a multivariable prediction model for Individual Prognosis Or Diagnosis (TRIPOD) framework for prediction model development (Collins et al. 2015:2; Page et al. 2021:2).

The protocol was registered prospectively in PROSPERO (Booth et al. 2012:148). The PROSPERO number ID: CRD420251026501.

### Eligibility

#### Inclusion criteria

Studies were eligible if they met the following criteria:

- included adults aged 18 years and above in South Africa.
- investigated risk factors or predictors associated with hypertension.
- defined hypertension as systolic blood pressure ≥ 140 mmHg, diastolic blood pressure ≥ 90 mmHg or current use of antihypertensive medication.
- used cross-sectional, case–control, cohort or interventional designs that reported relevant associations between predictors and hypertension.
- provided sufficient data to permit extraction or calculation of effect estimates for synthesis.

#### Exclusion criteria

Studies were excluded if they:

- were conducted outside South Africa, unless South African data could be clearly separated.
- focused on cardiovascular disease generally without a distinct analysis of hypertension.
- reported no usable effect measure or insufficient data for synthesis.
- were reviews, editorials, commentaries, conference abstracts or methodological papers without primary data.
- duplicated the same dataset without providing additional relevant information, in which case the most suitable report was retained.

### Search strategy

The search strategy was developed in consultation with an information specialist. Search terms were derived from key concepts related to hypertension, risk factors, prediction and South Africa. Both free-text terms and indexed subject headings were used where appropriate. Boolean operators such as AND and OR were applied to combine search concepts. The final search strategy was adapted to the syntax requirements of each database. The databases searched were PubMed, Scopus, Academic Search Complete, MEDLINE, Web of Science and African Journals (SABINET). Searches were conducted from January 1990 to March 2025. Where relevant, reference lists of included studies were also examined to identify additional eligible records.

### Search terms

The search terms covered the following major concepts:

- hypertension or high blood pressure.
- risk factors, predictors, determinants, associated factors
- South Africa.
- systematic combinations relevant to epidemiological association studies.
- A representative search strategy for PubMed is provided in the supplementary material.

### Study selection

All records retrieved from the database searches were exported to a reference management system for storage and deduplication before screening. Duplicate records were removed prior to selection. Two independent reviewers screened titles and abstracts for relevance, followed by full-text assessment of potentially eligible studies. The reviewers were MN and a trained research assistant. Disagreements were resolved through discussion and consensus. Reasons for exclusion at the full-text stage were documented and presented in the PRISMA flow diagram.

### Data extraction

For each eligible study, data were extracted using a predesigned extraction form. Extracted information included study design, year of publication, sample size, population characteristics, province or geographic setting, hypertension definition, predictor variables and effect estimates. Data extraction was undertaken by one reviewer and verified by a second reviewer to ensure consistency and accuracy.

### Quality appraisal

Methodological quality of the included studies was assessed using the Newcastle-Ottawa Scale for cohort and case–control studies (Wells et al. 2000), with appropriate adaptation for cross-sectional studies. This tool was selected because the review included observational study designs commonly assessed using Newcastle-Ottawa criteria. Quality appraisal was conducted after final study selection, and the findings were summarised narratively and in tabular form.

### Data analysis and presentation

Descriptive analysis was first undertaken to summarise the characteristics of the included studies, including the number of studies, study design distribution, publication years, provinces represented and notable predictors.

These descriptive findings are presented in Table 1. Meta-analysis was then conducted for eligible predictors using random-effects models based on log odds ratios and restricted maximum likelihood estimation. Between-study heterogeneity was assessed using I^2^ and τ^2^ statistics. Sensitivity analysis included exclusion of outliers and subgroup analysis by study design where appropriate. Small-study effects were assessed using Egger’s test when at least ten studies contributed to a pooled estimate. The synthesised pooled evidence was then used to inform the development of a clinical prediction model. Additional analyses included calculation of risk weight, geotemporal trend and predictor interaction density. Certainty of evidence was assessed using a modified GRADE framework and incorporated into the weighting process.

**TABLE 1:** Characteristics of included studies on hypertension in South Africa and Africa.

Authors (year)	Hypertension definition	Age	Sex	Study design	Sample size, populations and setting	Province	Race	Notable predictors or associations
Chidumwa et al. (2024)	≥ 140/90 mmHg	Adults ≥ 15 years	Male and female	Cross-sectional	18 024, community-based	KwaZulu-Natal	Black South Africans	Age, BMI, alcohol
Chikafu and Chimbari (2021)	≥ 140/90 mmHg or antihypertensive medication	Adults (≥ 18 years, mean: 45)	Male and female	Cross-sectional	131 adults, community-based	KwaZulu-Natal	Black South Africans	Age, obesity, alcohol use, family history
Du Plessis et al. (2022)	BP ≥ 140/90 mmHg (average of 3 measurements)	Adults (≥ 20 years, mean: 43)	Male and female	Cross-sectional, household survey	1995, community-based	North-West	Black South Africans	BMI, central obesity, smoking, alcohol, low education
Govender and Naidoo (2025)	Self-reported or measured ≥ 140/90 mmHg	Adults (≥ 18 years)	Male and female	Cross-sectional, health survey	400, hospital-based	KwaZulu-Natal	Black South Africans	Older age, male sex, diabetes, obesity
Grimsrud et al. (2009)	≥ 140/90 mmHg or antihypertensive medication	Adults ≥ 18 years	Male and female	Cross-sectional	4351, community-based	National	Mixed ancestry	BMI, ART duration, age
Gupta et al. (2023)	≥ 140/90 mmHg (measured)	18–65 years (mean: 42)	Male and female	Cross-sectional, urban cohort	4799	National	Nationally representative	Age, obesity, inactivity, family history
Mabhida et al. (2022)	≥ 140/90 mmHg	Adults ≥ 18 years	Male and female	Case-control	442, community-based	Eastern Cape	Black South Africans	Age, BMI, genetics
Madela et al. (2023)	≥ 140/90 mmHg	Adults ≥ 18 years	Male and female	Cross-sectional	7303, community-based	KwaZulu-Natal	Black South Africans	Age, BMI, family history
Masilela et al. (2020)	≥ 140/90 mmHg	Adults ≥ 18 years	Male and female	Cross-sectional	329, hospital-based	Mpumalanga	Black South Africans	BMI, alcohol, smoking
Matshazi et al. (2021)	≥ 140/90 mmHg or antihypertensive use	Adults (mean: 49)	Male and female	Cross-sectional, molecular and clinical	1456, community-based	Western Cape	Mixed ancestry and Black South Africans	Age, sex, BMI, microRNA biomarkers
Monakali et al. (2018)	≥ 140/90 mmHg or on treatment	Adults ≥ 18 years	Male and female	Cross-sectional	203, hospital-based	Eastern Cape	Black and mixed-race	Obesity, age, female sex
Naidoo et al. (2019)	Elevated BP defined by standardised measurement at age 23; linked to earlier BP trajectories (exact cut-off not explicitly stated in abstract)	23 years	49% male, 51% female	Longitudinal birth cohort (BT20)	1540, community-based	Gauteng	Black South Africans	Higher early-life linear growth ↑ risk; high childhood or adolescent BP trajectories → 4–5 increased adult risk
Nkeh et al. (2002)	≥ 140/90 mmHg	Adults ≥ 18 years	Male and female	Case-control	576, hospital-based	Gauteng	Black South Africans	Age, BMI, alcohol
Nkosi et al. (2021)	≥ 140/90 mmHg	Adults ≥ 18 years	Male and female	Cross-sectional	2397, community-based	Gauteng and North West	Black South Africans	Obesity, age, low physical activity
Ntlebi et al. (2020)	≥ 140/90 mmHg	Adults ≥ 18 years	Male and female	Cross-sectional	361, community-based	Gauteng	Mixed	BMI, age, diet
Ntuli et al. (2015)	≥ 140/90 mmHg	Adults ≥ 18 years	Male and female	Cross-sectional	1281, community based	Limpopo	Black South Africans	Age, diabetes, BMI
Owolabi et al. (2017)	≥ 140/90 mmHg	Adults ≥ 18 years	Male and female	Cross-sectional	998, hospital-based	Eastern Cape	Black South Africans	Age, BMI, male sex
Peer et al. (2025)	≥ 140/90 mmHg	Adults ≥ 18 years	Male and female	Cross-sectional	460, community-based	Western Cape	Black South Africans	Age, BMI, smoking
Peltzer and Phaswana-Mafuya (2013)	≥ 140/90 mmHg	Adults ≥ 50 years	Male and female	Cross-sectional	3840, community-based	National	Black, Coloured, Indian, White	Age, BMI, sex
Sharma et al. (2021)	≥ 140/90 mmHg	Adults ≥ 18 years	Male and female	Cross-sectional	487, community survey	Eastern Cape	Black South Africans	Age, BMI, alcohol
Sharma et al. (2024)	≥ 140/90 mmHg	Adults ≥ 18 years	Male and female	Case-control	487, hospital-based	Eastern Cape	Black South Africans	Age, BMI, WHR, Blood glucose
Steyn et al. (2008)	≥ 140/90 mmHg	Adults ≥ 18 years	Male and female	Cross-sectional	12 952, community-based	National	Black, Coloured, Indian, White	Age, BMI, low SES
Ware et al. (2019)	≥ 140/90 mmHg	Adults ≥ 18 years	Male and female	Cross-sectional	1847, community-based	National	Black South Africans	Age, BMI, male sex
Akpa et al. (2022)	SBP ≥ 140 or DBP ≥ 90 mmHg, or previous diagnosis or antihypertensive use	Mean 53.0 ± 10.7 years; adults ≥ 18	Male and female (45.9% male)	Secondary data analysis of cross-sectional survey	16 445, community-based	Multi-country (includes provinces across five African nations)	Indigenous Africans	Sufficient (OR 0.80) and high (OR 0.81) vegetable consumption protective
Ayo-Yusuf and Ayo-Yusuf (2008)	BP ≥ 160/95 mmHg and/or antihypertensive use	Adults 25–70 years	Male and female (60.6% female)	Secondary data analysis of cross-sectional survey	9098, community-based	National	Predominantly Black South Africans (75%)	Complete edentulousness significantly associated with hypertension (OR 1.35, 95% CI 1.02–1.78)
Candy et al. (2000)	DBP > 90 mmHg or 24-h ABPM > 85 mmHg (after washout)	Adults; hypertensives from clinics, normotensives from community	Male and female	Case-control genetic association study	192, hospital-based	Gauteng	Black South Africans	Arg16 homozygotes linked to lower BMI in hypertensives (*p* = 0.007); Glu27 polymorphism rarer (17%)
George et al. (2016)	SBP ≥ 140 or DBP ≥ 90 mmHg, or antihypertensive use at follow-up	Women, 29–53 years at baseline (mean 40 years), followed 8.3 years	Female only	Prospective cohort (baseline 2002–03, follow-up 2011–14)	478, institution-based	Gauteng	Black South African women	Central adiposity (waist circumference, trunk fat) predicted hypertension more strongly than total adiposity
Lategan, Van den Berg and Walshet (2014)	SBP ≥ 140 or DBP ≥ 90 mmHg, or on antihypertensive medication	25–64 years (adults)	Male and female	Cross-sectional (Assuring Health for All in Free State substudy)	339, community-based	Free State	Black South Africans (urban, transitioning community)	Positive correlation between BP and BMI, WHtR, BAI; HIV inversely correlated with adiposity indices
Kandala et al. (2021)	BP ≥ 140/90 mmHg or self-reported diagnosis or antihypertensive use	≥ 15 years	Male and female	Secondary analysis of cross-sectional surveys	18 231, community-based	National (all 9 provinces)	Black, Coloured, White, Indian or Asian South Africans	Higher odds with male sex, coloured group, urban residence, high cholesterol, age, BMI, waist circumference
Magodoro et al. (2022)	SBP ≥ 140 or DBP ≥ 90 mmHg, or antihypertensive use	≥ 15 years (median 36 years)	Male and female	Nationally representative cross-sectional survey (DHS 2016)	6420, community-based	National	Black, White, Coloured, Indian or Asian South Africans	Female HIV-negatives had 5 mmHg higher SBP than HIV+; HIV not associated with worse cardiometabolic profile
Mphekgwana et al. (2020)	SBP ≥ 140 and DBP ≥ 90 mmHg	≥ 18 years (adults 18–101 years)	Male and female	Cross-sectional (population-based)	2256, community-based	Limpopo	Black South Africans	Semi-urban women: PA, family history, fruit intake, salty food, meat; rural women: WHR, diabetes, salty food; men: fried food, low fruit intake, WHR
Wandai et al. (2020)	SBP > 140 or DBP > 90 mmHg, or antihypertensive medication use	≥ 15 years	Male and female	Panel survey multilevel analysis (NIDS waves 2008–2017)	93 787, community-based	National (district-level analysis across nine provinces)	Black, Coloured, White, Indian or Asian South Africans	Age, BMI, race strong predictors; also influenced by alcohol, smoking, education, income, season
Nguyen et al. (2022)	SBP ≥ 140 or DBP ≥ 90 mmHg, or self-reported diagnosis	Women, 18–40 years	Female only	Cross-sectional (baseline of Rape Impact Cohort Evaluation study)	1742, hospital-based	KwaZulu-Natal	Black South African women	Frequent NPSV, SH, physical and emotional IPV linked to hypertension; depression, PTSD, binge drinking mediated associations.
Ntlahla et al. (2021)	SBP ≥ 140 or DBP ≥ 90 mmHg (pre-HTN: SBP 120–139 or DBP 80–89)	18–25 years (youth)	Male and female	Cross-sectional quantitative study	151, university community	Eastern Cape	South African youth (Black)	Zonulin (gut permeability marker) higher in hypertensives; positively correlated with SBP in women, and obesity indices
Pisa et al. (2018)	SBP ≥ 140 or DBP ≥ 90 mmHg, or on hypertension medication	Middle-aged adults (mean 40–60 years)	Male and female	Cross-sectional, AWI-GEN Soweto site	2008, community-based	Gauteng	Urban Black South Africans	All adiposity indices associated with hypertension; FM/FFSTM ratio strongest predictor; WC significant predictor in multivariate models
Patel et al. (2016)	SBP ≥ 140 or DBP ≥ 90 mmHg, or on antihypertensive medication	Adults 20–79 years	Male and female	Cross-sectional, multi-country (including South Africa)	1088, community-based	Western Cape (Cape Town site)	Multi-ethnic (focus on South Africans within global sample)	BMI and WC both predicted hypertension; associations weakest in South Africa compared to other regions
Raphadu et al. (2023)	Children and/or adolescents: NHLBI cut-offs by age/sex/height; Adults: SBP ≥ 130 or DBP ≥ 80 (2017 ACC/AHA)	Tracked from childhood (5–12 years in 1999) to young adulthood (18–30 years in 2015)	Male and female (164 women, 161 men)	Longitudinal cohort (Ellisras Longitudinal Study)	325, school-based	Limpopo	Rural Black South Africans	Sodium-to-potassium ratio stronger predictor than Na or K alone
Schutte et al. (2012)	Optimal BP ≤120/80; hypertension ≥ 140/90 mmHg	Adults ≥ 30 years	Male and female	Prospective cohort (PURE study, 2005–2010)	478, community-based	North West	Black South Africans	Smoking, alcohol intake, waist circumference, γ-GT predicted HT; HIV infection inversely associated with BP rise
Solomons, Kruger and Puoane (2020)	SBP ≥ 140 or DBP ≥ 90 mmHg	Adults 32–81 years	Male and female	Secondary analysis of a cross-sectional study (PURE-Langa site, 2010)	454, community-based	Western Cape	Urban Black South Africans	Poor adherence to dietary guidelines linked to higher BP; added sugar intake correlated with SBP in women
Wand et al. (2022)	SBP ≥ 130 or DBP ≥ 80 mmHg, or diagnosis and medication (2017 ACC/AHA)	≥ 15 years (median age 32)	Male and female	Nationally representative cohort (SA-NIDS 2008–2017)	80 270, community-based	National (nine provinces)	Black South Africans	Age, low education, obesity, high WC, smoking, alcohol, low exercise; urban residence predictive in men
Rudasingwa, Cho and Ishimwe (2024)	SBP ≥ 140 or DBP ≥ 90 mmHg, or on antihypertensive treatment	≥ 15 years (adults)	Male and female	Secondary analysis of cross-sectional survey	3172, community-based	National (with provincial and spatial hotspot analysis)	South Africans (multi-province)	Older age, unmarried, diabetes or pre-diabetes, increased risk; education protective; Limpopo and North West protective
Ntenda et al. (2022)	Self-reported hypertension (doctor or nurse told respondent they have HTN)	Women ≥ 20 years (mean 40.7 ± 18.4 years)	Female only	Secondary analysis of a cross-sectional survey (SADHS 2016)	6027, community-based	National (all nine provinces analysed with provincial variation)	Predominantly Black or African (84.5%), White (4.3%), Coloured or other (11.2%)	Older age; never married; no insurance protection; Black or African ethnicity; overweight or obese perception; higher risk in Western Cape, Eastern Cape, Northern Cape, Free State, North West, Gauteng, Mpumalanga compared to Limpopo
Gass et al. (2010)	Self-report	Women, ≥ 18 years	Female	Cross-sectional	1229, mixed-community and hospital-based	National	Mixed	None
Jamshidi, Khodayari Bavil and Egal (2019)	HTN ≥ 130/80 mmHg	Mean = 72.8 (8.66)	Male and female	Cross-sectional	275, elderly or faith-based day-care centre	Gauteng	Black	Hb and ferritin
Matshazi et al. (2021)	HTN ≥ 140/90 mmHg	Mean = 42.6 years	Male and female	Secondary analysis of a cross-sectional study	881, community-based	Western Cape	N/A	miR-novelchr1_36178 and miR-novel-chr15_18383
Mazenda, Katiyatiya and Sari (2025)	Self-report	N/A	Male and female	Cross-sectional	3278, community-based	Gauteng	N/A	Protein-based diet
Modjadji et al. (2022)	HTN ≥ 140/90 mmHg	≥ 60 years	Male and female	Cross-sectional	350, community-based	Limpopo	Black	BMI, WC, WHtR
Motlhale and Ncayiyan (2019)	HTN diagnosed by a health care provider	Adults ≥ 18 years	Male and female	Cross-sectional	28 007, community-based	Gauteng	Mixed	Migration
Sekgala et al. (2022)	HTN ≥ 130/85 mmHg	Males ≥ 20 years	Males	Cross-sectional	185, community-based	Western Cape	N/A	Percentage of protein consumed
Nguyen et al. (2022)	BP ≥ 140/90 mmHg or previous diagnosis	Median age of 24 years	All female	Cross-sectional	1797, rape-exposed and non-rape-exposed participants	KwaZulu-Natal	Predominantly Black South African	Childhood maltreatment (sexual, physical, emotional abuse, neglect)
Chidumwa et al. (2021)	HTN ≥ 140/90 mmHg +/or antihypertensive	Median 56 years	Male and female	Nested cross-sectional spatial modelling	2761, community-based	National sample (all nine provinces; highest in KwaZulu-Natal and Gauteng)	Mixed South African population (all major ethnic groups)	Hypertension and diabetes co-occur; higher shared spatial risk in KwaZulu-Natal and central Gauteng
Jacobs et al. (2019)	SBP ≥ 140 mmHg or DBP ≥ 90 mmHg, or self-reported antihypertensive medication use	≥ 30 years	Men and women	Cohort	2010, community-based	North West	Black South Africans	PAI-1act, 4G/4G genotype, 5G/5G Genotype, WC
Naidoo, Fabian and Norris (2023)	≥ 140/90 mmHg	28 years	52% female	Cross-sectional	933, community-based	Gauteng province (Soweto)	Predominantly Black South African	Male, BMI, hyperuricaemia, albuminuria (in men), and self-reported history of gestational hypertension (in women).
Renta et al. (2022)	Self-reported and measured hypertension; measured ≥ 140/90 mmHg	Mean = 60.9 (13.1) rural and 59.9 (11.9) urban	Male and female	Nested cross-sectional, WHO-SAGE	4227, community-based	National sample (all nine provinces)	All provinces	No rural/urban interaction; country differences in social capital-hypertension link

WHO, World Health Organization; BMI, body mass index; BP, blood pressure; ART, antiretroviral therapy; OR, odds ratio; CI, confidence interval; HIV, human immunodeficiency virus; SABSSM, South African Social Attitudes Survey; WHR, waist-to-hip ratio; SES, socioeconomic status; SBP, systolic blood pressure; DBP, diastolic blood pressure; SIREN, Stroke Investigative Research and Educational Network; AWI, Africa Wits-INDEPTH Partnership for Genomic Studies; ABPM, ambulatory blood pressure monitoring; WHtR, waist-to-height ratio; BAI, body adiposity index; DHS, Demographic and Health Survey; NPSV, non-partner sexual violence; SH, sexual harassment; IPV, intimate partner violence; PTSD, post-traumatic stress disorder; FM/FFSTM, fat mass/fat-free soft tissue mass ratio; WC, waist circumference; NHLBI, National Heart, Lung, and Blood Institute; PURE, Prospective Urban and Rural Epidemiological Study; HT, hypertension; HTN, hypertension; Hb, haemoglobin.

### Additional analysis

We calculated risk weight (RW), risk responsiveness, geotemporal trend and predictor interaction density (PI) as per these studies (Ibeneme et al. 2024:1402; Nweke & Mshunqane 2025:405; Nweke et al. 2025a, 2025b:282). To stabilise the risk estimate, we used the certainty of evidence as a global shrinkage factor to minimise overfitting (Steyerberg & Harrell 2016:245–247). Certainty was graded using a modified GRADE rubric (Page et al. 2021:n71), with weights of 1.0 (high), 0.75 (moderate), 0.5 (low) and 0.25 (very low). The certainty-adjusted RW was determined by multiplying Rw by the certainty weight. The critical risk threshold was established following (Ibeneme et al. 2024:1402; Nweke & Mshunqane 2025:405; Nweke et al. 2025a, 2025b:282).

### Predictor governance

Predictors were managed using RW and evidence certainty. Pooled log odds ratios were mapped to a directed acyclic diagram to determine the predictor interaction density (PID). Predictors were retained only when both causal plausibility and evidentiary strength were present.

### Ethical considerations

Ethical clearance to conduct this study was obtained from the Faculty of Health Sciences Research Ethics Committee of Durban University of Technology (No. IN-REC-025/25). Informed consent was not applicable because this review used data extracted from previously published studies and did not involve direct contact with human participants. The included primary studies had already undergone their respective ethical review processes as reported by their original authors.

## Results

### Search results and characteristics of studies

A total of 62 003 records were identified through database searching. After removal of 28 971 duplicates, 33 032 records remained for title and abstract screening. Of these, 32 939 were excluded. Ninety-seven reports were sought for retrieval, four could not be retrieved, and 91 full-text articles were assessed for eligibility. Thirty-five reports were excluded following the full-text review. Ultimately, 54 studies, comprising six cohort, three case–control and 45 cross-sectional studies, were included in the review and meta-analysis (Figure 1). Fifteen studies were national in scope and covered all nine provinces of South Africa. The included studies spanned multiple provinces and publication years, and together provided evidence on demographic, biological, behavioural, environmental and geographic predictors of hypertension. Study characteristics are presented in Table 1.

**FIGURE 1:**
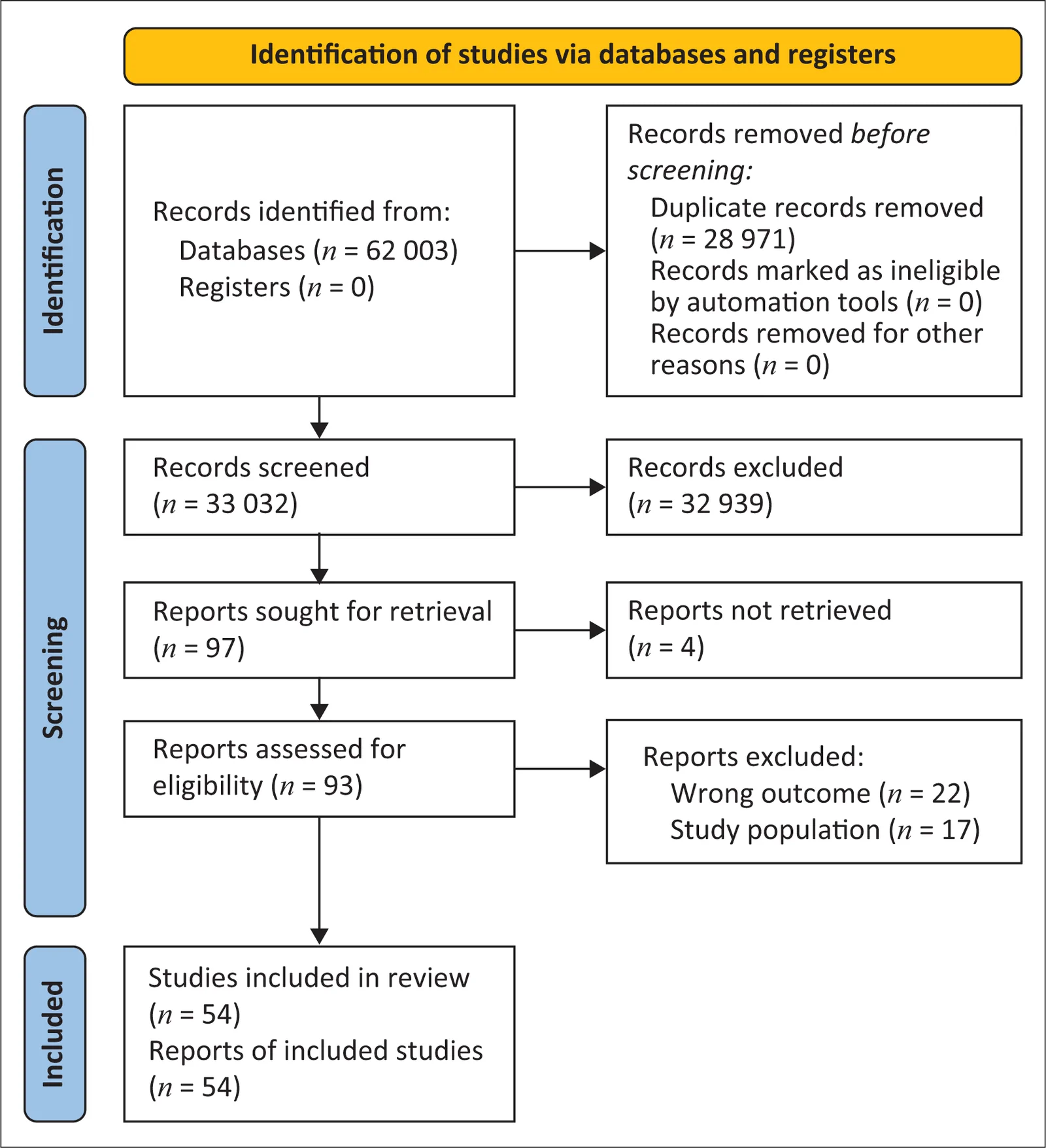
Preferred Reporting Items for Systematic Reviews and Meta-Analyses 2020 flow diagram.

### Meta-analysis and certainty of evidence

Among the eighteen factors eligible for meta-analysis, ten were found to have a statistically significant link to hypertension. The most notable associations were with dyslipidaemia (OR: 3.79, CI 1.84–7.80), age over 50 years (OR: 3.49, CI 1.82–6.70), diabetes (OR: 2.35, CI 1.89–2.91) and obesity (OR: 2.57, CI 1.86–3.53). After evaluating the evidence, dyslipidaemia (OR 3.79, low) and age over 50 (OR 3.49, low) were categorised as having a strong association, albeit with low certainty. Diabetes (OR 2.57, moderate) and BMI (obesity, OR 2.35, moderate), along with exposure to gold-mine dust (OR 2.35, moderate), were also strongly linked to hypertension, supported by moderate evidence. Secondary education (OR 0.64, very low) and tertiary education (OR 0.56, very low) appeared to have protective effects, though the certainty was very low. Low intake of fruits and vegetables (OR 1.16, low) and living in urban areas (OR 1.20, very low) were close to the baseline, indicating minimal association strength and low certainty. Family history (OR 1.45, low) and residing in regions other than Limpopo (OR 2.12, low) showed modest associations with low certainty (Table 2 and Table 3).

**TABLE 2:** Meta-analysis of factors associated with hypertension in South Africa.

Factors	Ref	Exposure	Odds ratio	95% CI	I^2^ (%)	Egger’s test *p*	k	Ri	GRADE	Rw	Rw-adjusted	cRW	cRW-adjusted	GTT
Age (years)	< 50	> 50	3.49	1.82–6.70	97.8	0.263	7	0.86	0.50	3.00^U^	1.50	3.00	1.50	0.93
Education	None	Tertiary	0.56	0.39–0.80	95.0	0.810	12	0.75	0.25	1.34^L^	0.34	4.34	1.84	0.88
BMI	Normal	Obesity	2.35	1.89–2.91	88.3	0.630	17	0.88	0.50	2.07^M^	1.04	6.41	2.87	0.94
Family History of HTN	No	Yes	1.45	1.22–1.71	48.8	0.580	4	0.75	0.50	1.09^M^	0.55	7.50	3.42	0.71
Diabetes	No	Yes	2.57	1.86–3.53	89.1	0.180	15	0.80	0.75	2.06^U^	1.55	9.56	4.96	0.90
Dyslipidemia	No	Yes	3.79	1.84–7.80	90.5	0.420	4	0.75	0.50	2.84^M^	1.42	12.40	6.38	0.71
Fruits and vegetables	Sufficient	Low intake	1.16	1.02–1.33	51.7	0.830	5	0.40	0.50	0.46^L^	0.23	12.86	6.61	0.70
Gold-mine dust	Unexposed (> 5 km away)	Exposed (< 5 km away)	2.35	1.70–3.25	78.0	0.600	3	1.00	0.75	2.35^U^	1.76	15.21	8.37	0.57
Settlement	Rural	Urban	1.20	1.00–1.43	87.4	0.770	10	0.40	0.25	0.48^L^	0.12	15.69	8.49	0.70
Region/province	Limpopo	Others	2.02	1.70–2.39	0.00	0.683	5	1.00	0.50	2.02^M^	1.01	17.71	9.50	1.00

BMI, body mass index; HTN, hypertension; I^2^, heterogeneity; k, number of studies involved in the synthesis of a given risk factor; Ri, risk responsiveness; RW, risk weight; GTT, geotemporal trend; U, upper class high (> 1.48); M, middle class: 0.39–1.48; L, lower class: ≤ 0.39.

**TABLE 3:** Grading of evidence for risk factors of hypertension in South Africa.

Risk factor (population: adults in South Africa)	Effect	OR, 95% CI	Certainty of evidence (GRADE)	Reasons for downgrade or upgrade	Interpretation
Age > 50	3.49	1.82–6.70	⨁⨁ ◯ ◯ Low	Imprecision −1; Large effect +1	Older age strongly associated with hypertension; evidence limited by imprecision.
Education (secondary)	0.64	0.48–0.86	⨁ ◯ ◯ ◯ Very low	Inconsistency −1	Secondary education may be protective; inconsistent findings.
Education (tertiary)	0.56	0.39–0.80	⨁ ◯ ◯ ◯ Very low	Imprecision −1	Tertiary education likely protective but imprecise estimates.
BMI (overweight)	1.58	1.42–1.76	⨁⨁ ◯ ◯ Low	None	Overweight modestly increases hypertension risk.
BMI (obesity)	2.35	1.89–2.91	⨁⨁⨁ ◯ Moderate	Large effect +1	Obesity strongly increases hypertension risk; upgraded for large effect.
Family history of HTN	1.45	1.22–1.71	⨁⨁ ◯ ◯ Low	None	Family history minimally increases risk; no large effect upgrade.
Diabetes	2.57	1.86–3.53	⨁⨁⨁ ◯ Moderate	Large effect +1	Diabetes strongly associated with hypertension; upgraded for large effect.
Dyslipidaemia	3.79	1.84–7.80	⨁⨁ ◯ ◯ Low	Imprecision −1; Large effect +1	High risk but downgraded for imprecision.
Fruits and vegetables (low)	1.16	1.02–1.33	⨁⨁ ◯ ◯ Low	None	Minimal effect; evidence remains low certainty.
Gold-mine dust	2.35	1.70–3.25	⨁⨁⨁ ◯ Moderate	Large effect +1	Occupational dust exposure increases hypertension risk; upgraded for large effect.
Settlement (urban)	1.20	1.00–1.43	⨁ ◯ ◯ ◯ Very low	Indirectness −1	Urban residence shows a small effect; downgraded for indirectness.
Region (others vs Limpopo)	2.12	1.78–2.51	⨁⨁ ◯ ◯ Low	Indirectness −1; Large effect +1	Regional differences suggest higher risk outside Limpopo; upgrade for large effect offsets indirectness.

BMI, body mass index; HTN, Hypertension.

### Model parameters

Among the ten identified risk factors for hypertension, age over 50 years (Rw: 3.00, GTT: 0.93), dyslipidaemia (Rw: 2.84, GTT: 0.71) and exposure to gold-mine dust (Rw: 2.35, GTT: 0.57) were classified as high risk. In contrast, obesity (Rw: 2.07, GTT: 0.94), geographic location (Rw: 2.02, GTT: 1.0), along with family history of hypertension, educational background and low intake of fruits and vegetables, were categorised as lower risk. Most risk factors demonstrated substantial geotemporal coverage (GTT: 0.7–1.0). Environmental factors showed the lowest interaction density (Figure 2). The critical risk threshold at the 75th percentile was determined to be 7.93 (Table 2).

**FIGURE 2:**
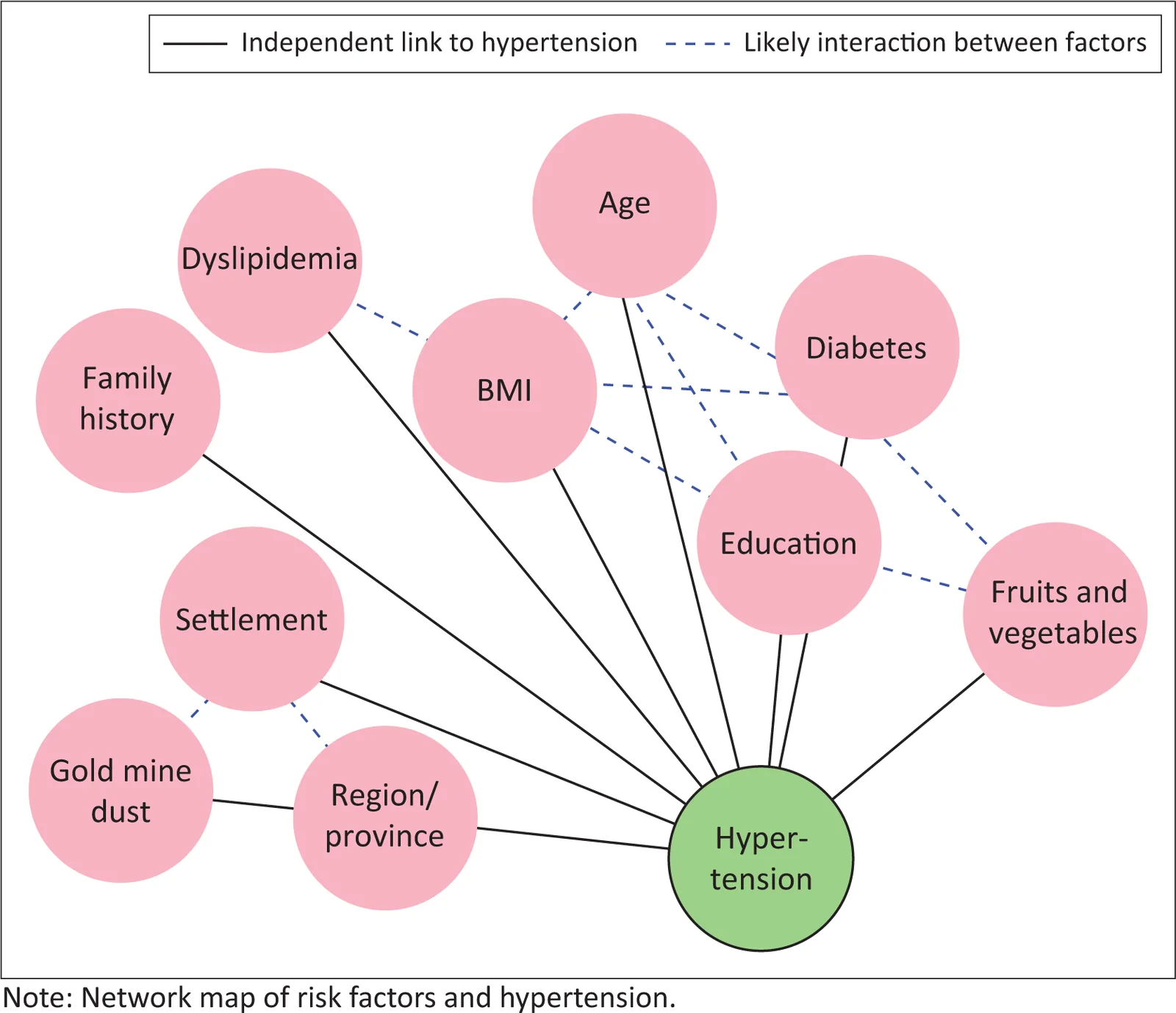
Directed acyclic diagram showing the interaction of hypertension risk factors.

The results show that there were thirty-seven possible ways in which the ten risk factors could combine to reach the critical risk threshold (CRT) (Online Appendix 1, Table 1-A1 and Table 2-A2). Upon examination of the hypothetical interaction among the ten risk factors of hypertension in South Africa (Figure 2), result shows that model 37 was robust and the most parsimonic model with minimal interaction density (Supplementary File 1). Therefore, the predictive algorithm was set forth in Table 4.

**TABLE 4:** Clinical prediction algorithm for hypertension in South Africa.

Factors	Reference	Exposure or protective factor	Point
Age (years)	< 50	> 50	+1.50
Education	None/primary	Tertiary	−0.84
	None/primary	Secondary	−0.29
BMI	Normal	Obesity	+1.04
	Normal	Overweight	+0.42
Family history of HTN	No	Yes	+0.55
Diabetes	No	Yes	+1.55
Dyslipidaemia	No	Yes	+1.42
Fruits and vegetables	Sufficient	Low intake	+0.23
Gold-mine dust	Unexposed (> 5 km away)	Exposed (< 5 km away)	+1.76
Settlement	Rural	Urban	+0.12
Region/province	Limpopo	Others	+1.01

BMI, body mass index; HTN, Hypertension.

Low risk → values ≤ 3.01 (at or below the 25th percentile).

Moderate risk → values > 3.01 up to 5.67 (between the 25th and 50th percentiles).

Elevated risk → values > 5.67 up to 7.93 (between the 50th and 75th percentiles).

High risk → values > 7.93 (above the 75th percentile).

## Discussion

This study first synthesised pooled evidence on risk factors associated with hypertension in South Africa and then used these findings to develop a context-specific clinical prediction model. The meta-analysis identified older age, obesity, diabetes, dyslipidaemia, gold-mine dust exposure and certain geographic factors as significant predictors of hypertension, while education appeared protective. These pooled estimates were subsequently translated into the algorithmic structure of the prediction model, enabling the meta-analytic findings to inform a practical risk stratification tool. Overall, the results provide a more robust summary of the South African evidence base than any single primary study and highlight the importance of both conventional metabolic predictors and context-specific structural exposures.

The pooled findings are broadly consistent with previous epidemiological evidence showing that hypertension risk increases with age, obesity, diabetes and dyslipidaemia (Forouzanfar et al. 2017:165–174; Mills et al. 2020:223–237; Roth et al. 2020:2982–3021). The strong association observed for age over 50 years reflects the well-established relationship between ageing, arterial stiffness and cumulative cardiovascular risk exposure (Franklin et al. 1997:308–315; Lakatta & Levy 2003:139–146; North & Sinclair 2012:1097–1108). Similarly, the associations for obesity and diabetes affirm the central role of metabolic dysfunction in hypertension (Mills et al. 2020:223–237; Stringhini et al. 2017:1229–1237). The positive association with gold-mine dust exposure is especially important in the South African context, where occupational and environmental risk factors may be under-recognised in conventional risk models (Stanaway et al. 2018:1923–1944). The observed protective association for education is also plausible, as higher educational attainment may be linked to improved health literacy, healthier behaviours and better access to healthcare (Cutler & Lleras-Muney 2010:1–28; Kaplan & Keil 1993:1973–1998). Nevertheless, some predictors were supported by low or very low certainty evidence, which warrants caution in interpretation (Guyatt et al. 2008:924–926; Page et al. 2021:71).

Our proposed hypertension risk algorithm, developed using South African data, includes factors such as age, BMI, family history of hypertension, diabetes, dyslipidaemia, low consumption of fruits and vegetables, exposure to occupational dust, type of settlement and region or province, with education considered a protective element.

The weighting system gives the highest values to gold-mine dust (Rw = 1·76), diabetes (1·55), age over 50 (1·50), and dyslipidaemia (1·42), while BMI, family history, and regional factors have smaller contributions, and secondary or tertiary education receives negative points. This setup captures both well-known metabolic influences and specific exposures relevant to South Africa (Mayosi et al. 2009:934–947; Shisana et al. 2013).

When compared to international prediction models, the differences in factor selection are significant. The Framingham hypertension risk score, originally developed in the United States, prioritises baseline systolic and diastolic blood pressure, followed by age, parental history, BMI and smoking (D’Agostino et al. 2008:743–753). In contrast, our model omits baseline blood pressure and smoking, thus reducing the data requirements in primary care settings. Global cardiovascular risk models, like the World Health Organization (WHO) Cardiovascular disease (CVD) risk charts and the QRISK series, aim to predict 10-year CVD events rather than new cases of hypertension, typically including age, sex, smoking, blood pressure, lipids and diabetes (Hippisley-Cox, Coupland & Brindle 2017:2099; Kaptoge et al. 2019:e1332–e1345).

These tools are well validated across various populations but do not account for environmental and occupational exposures that are particularly relevant in South Africa.

Locally, the only similar tool is the six-item hypertension risk score recently created for South Africa, which focuses on older age and adiposity but does not include environmental or geographic factors (Wand et al. 2022:451–461). National practice otherwise depends on the South African hypertension guideline and WHO HEARTS protocols, which categorise care by blood pressure level and absolute CVD risk rather than predicting new hypertension cases (Unger et al. 2020:1334–1357; WHO 2025). Our model’s inclusion of education, occupational exposure, settlement and provincial differences offers a level of contextual specificity not found in existing global or national tools.

The exclusion of sex and smoking from our final model sets it apart from most international algorithms, where these factors, though having a modest effect, are standard (D’Agostino et al. 2008:743–753; Hippisley-Cox et al. 2017:j2099). The choice to leave out baseline blood pressure also differentiates our approach from Framingham and QRISK, but it was necessary to create a simplified, office-friendly screening tool. The protective weighting of education aligns with the social gradient in hypertension observed in South African epidemiological studies (Shisana et al. 2013), while the positive weighting of gold-mine dust and urban settlement highlights the structural determinants of hypertension risk in the country. The resulting score provides discrimination using factors that are both measurable in routine care and directly relevant to national health priorities.

The weighting scheme is consistent with epidemiological findings that metabolic risk factors are the primary indicators of hypertension while also emphasising the additional explanatory power of context-specific exposures (Mayosi et al. 2009:934–947). Unlike the Framingham score, which prioritises blood pressure and age (D’Agostino et al. 2008:743–753), our model gives equal importance to diabetes, dyslipidaemia and exposure to occupational dust. These distinctions underscore the necessity for models tailored to specific contexts and suggest the importance of formal calibration against absolute risk estimates for comparison with global tools. To ensure generalisability, external validation across different provinces and among populations with and without mining exposure will be crucial. Despite this, the model’s feasibility and contextual relevance indicate a strong potential for its integration into South African primary care as a tool for screening and early prevention.

This study is not without limitations. Firstly, the evidence base was largely dominated by cross-sectional studies with relatively few cohort studies, which limits inferences about temporality and causation and increases susceptibility to residual confounding. Secondly, the certainty of evidence was low or very low for many predictors included in the model. Thirdly, although we applied a global certainty-based shrinkage to reduce overfitting, the algorithm has not yet undergone internal–external or external validation; its discrimination, calibration and clinical utility outside the derivation evidence base remain untested.

### Implications and recommendations

The findings of this review have both clinical and public health implications. Clinically, they suggest that hypertension risk assessment in South Africa should extend beyond conventional metabolic markers to include occupational, social and geographic determinants where relevant. From a public health perspective, the results support the need for more context-specific approaches to screening and prevention. There is also a need for internal validation using independent South African cohorts and external validation in other African populations.

For implementation research, pilot studies in primary care and occupational health settings are recommended to evaluate feasibility, acceptability and utility relative to current practice.

## Conclusion

This study synthesised pooled South African evidence on risk factors associated with hypertension and used these findings to inform development of a clinical prediction model. The pooled evidence showed that older age, obesity, diabetes, dyslipidaemia, gold-mine dust exposure and selected geographic factors were among the most important predictors, while education appeared protective. Based on these findings, a context-specific prediction algorithm was developed that incorporates metabolic, social, environmental and geographic factors relevant to South Africa. The model offers a potentially useful screening approach for primary care and prevention but requires internal and external validation before routine application.
